# Concomitant Use of Intravenous Remimazolam With Inhalation Anesthesia and Subsequent Emergence Delirium in Children: A Systematic Review and Meta-Analysis

**DOI:** 10.7759/cureus.80044

**Published:** 2025-03-04

**Authors:** Yukie Nitta, Yuji Kamimura, Akihiro Shiroshita, Kanta Kido

**Affiliations:** 1 Department of Dental Anesthesiology, Faculty of Dental Medicine and Graduate School of Dental Medicine, Hokkaido University, Sapporo, JPN; 2 Department of Systematic Reviewers, Scientific Research WorkS Peer Support Group (SRWS-PSG), Osaka, JPN; 3 Department of Anesthesiology and Intensive Care Medicine, Nagoya City University Graduate School of Medical Sciences, Nagoya City University, Nagoya, JPN; 4 Department of Medicine, Vanderbilt University School of Medicine, Nashville, USA

**Keywords:** emergence delirium, meta-analysis, pediatric anesthesia, remimazolam, systematic review

## Abstract

Emergence delirium (ED) is a typical postoperative complication in pediatric anesthesia, especially with inhalational agents. Remimazolam, a short-acting benzodiazepine, shows potential for reducing the occurrence of ED. Given limited evidence of its use in pediatric anesthesia, we evaluated the efficacy and safety of remimazolam by conducting a systematic review and meta-analysis.

MEDLINE, EMBASE, Cochrane Central Register of Controlled Trials, ClinicalTrials.gov, and International Clinical Trials Registry Platform (ICTRP) databases were explored for studies on remimazolam in pediatric anesthesia. The studies included were randomized controlled trials (RCTs), prospective and retrospective cohort studies, case series, and case reports. Eligible patients were pediatric patients as American Society of Anesthesiologists Physical Status I or II who underwent sevoflurane-based general anesthesia. Primary outcomes included ED and emergence time. Study quality was assessed using the Risk of Bias 2 tool, and evidence certainty was evaluated by the Grading of Recommendations, Assessment, Development, and Evaluation approach. Random-effects meta-analyses estimated pooled risk ratios (RRs).

Three RCTs (n = 310) were included. A 0.2 mg/kg remimazolam bolus may result in a large reduction in ED (RR 0.26, 95% confidence intervals (CIs) 0.16 to 0.44, I² = 0%; low certainty, three studies). Continuous infusion showed similar effects (RR 0.22, 95% CIs 0.08 to 0.60, low certainty, one study). Emergence times varied by dosage and administration method, with continuous infusion associated with prolonged emergence times (mean difference 5.7 minutes, 95% CIs 3.67 to 7.73, low evidence, one study). Evidence certainty ranged from very low to low, with the 0.2 mg/kg bolus rated very low.

The concomitant use of intravenous remimazolam with inhalation anesthesia may reduce the ED in pediatric patients. However, evidence on emergence times remains inconclusive. Anesthetists could potentially use remimazolam to reduce the ED in children after inhalation anesthesia, but further investigation regarding its efficacy and safety across diverse populations is warranted.

## Introduction and background

Emergence delirium (ED) in children is characterized by a sudden onset of disorientation, perceptual problems, and hyperactive motor behavior immediately after anesthesia recovery [1,2]. It is commonly observed in pediatric anesthesia, particularly with inhalational agents, and has an incidence rate ranging between 10% and 80% [1]. This condition often leads to temporary agitation and uncooperative behavior, potentially increasing the duration of patient stay in the post-anesthesia care unit (PACU).

A variety of pharmacologic strategies has been investigated to prevent ED in children, emphasizing the ongoing need for effective interventions [3]. Propofol has traditionally been widely used for this purpose, with its efficacy supported in diverse clinical contexts [4,5]. However, the formulation of propofol includes allergens, such as soybean and egg derivatives, restricting its use in patients with specific food allergies [6]. This creates a need for alternative ED prevention options, particularly for patients at risk of allergies, prolonged sedation, or other adverse effects.

Remimazolam is a recently introduced intravenous benzodiazepine with an ultra-short duration of action. It has proved to be advantageous in clinical practice, as it offers rapid onset and subsequent recovery. Results from recent randomized controlled trials (RCTs) indicate that remimazolam could help decrease the frequency of pediatric ED [7-9]. Studies have demonstrated that both single-bolus administration and continuous infusion of remimazolam are linked to a reduced incidence of ED compared to placebo despite variations in dosing and administration methods across trials. However, the limited sample sizes of individual RCTs limit the comprehensive assessment of adverse events.

To address these limitations, we conducted a systematic review and meta-analysis (SR/MA) examining the available data regarding the efficacy and safety of remimazolam in the prevention of ED.

We published this protocol in the Open Science Forum (https://osf.io/h8wgq/).

## Review

Methods

Compliance With Reporting Guideline

We used a systematic review protocol template (Appendix G). We followed the Preferred Reporting Items for Systematic Review and Meta-Analysis Protocols (PRISMA-P) 2015 [10] and the recommendations listed in the Cochrane Handbook [11]. 

Eligibility Criteria

This study aimed to address the research question concerning the effectiveness of the concomitant use of intravenous remimazolam with inhalation anesthesia and subsequent ED in children. Participants were defined as male or female pediatric patients aged 0 to 18 years with an American Society of Anesthesiologists Physical Status (ASA-PS) classification of I or II, who underwent general anesthesia using inhaled sevoflurane during surgery. We defined intervention as the administration of intravenous remimazolam (bolus or continuous infusion) during the perioperative period. We defined control as the administration of a placebo, no intervention, or usual care.

We included RCTs, prospective and retrospective cohort studies, case series, and case reports on the relationship between ED and intravenous remimazolam in children undergoing general anesthesia with inhaled sevoflurane. We did not apply language, country, or publication year restrictions. We excluded pediatric participants who were ASA-PS ≥ 3 and were scheduled for elective surgery under total intravenous anesthesia. We also excluded adult (over 18 years of age) participants. We considered both published and unpublished studies, including conference abstracts and correspondence.

Outcomes of Interest

The primary outcomes of interest focused on the incidence of ED (within 30 minutes in the PACU) and emergence time (during the follow-up period). We used the Pediatric Anesthesia Emergence Delirium (PAED) scale to assess ED [12]. ED was defined as a global PAED score ≥ 10. A score of 10 or greater indicates a sensitivity of 0.64 and a specificity of 0.14 [12]. The PAED scale includes five parameters: eye contact, purposeful behavior, awareness of surroundings, restlessness, and inconsolability. Items 1, 2, and 3 are scored on a scale ranging from 4, representing "not at all," to 0, representing "extremely so," with intermediate values of 3 for "a little," 2 for "a lot," and 1 for "very much." In contrast, items 4 and 5 are scored inversely. We defined emergence time as the interval between the termination of inhaled anesthetics and the opening of the eye upon verbal commands.

Secondary outcomes encompassed the duration of PACU stay (during follow-up), the severity of delirium (during follow-up), and all adverse events (during hospitalization). We defined the duration of PACU stay as the time elapsed from the patient’s arrival in the PACU to their discharge readiness. The peak PAED score was used as an indicator of delirium severity. Adverse events were classified according to the definitions provided by the original study authors.

Information Sources and Search Strategy

Our search was conducted across multiple databases, including MEDLINE (via PubMed), EMBASE (via Ovid), and the Cochrane Central Register of Controlled Trials (Cochrane Library). Additionally, we reviewed the World Health Organization International Clinical Trials Platform Search Portal (ICTRP) and ClinicalTrials.gov to capture both ongoing research and unpublished trials. The complete search strategy is provided in Appendix F. We checked the reference lists of international guidelines and eligible studies (including those awaiting classification) as well as articles citing the eligible studies (including those awaiting classification).

Selection Process

Two independent reviewers (YN and KK) assessed the titles and abstracts to identify potentially relevant studies and examined the full texts to confirm their eligibility. If relevant data were missing, we contacted the original authors. Any disagreements between the two reviewers were resolved through discussion, and when consensus could not be reached, a third reviewer arbitrated (YK).

Data Collection Process

Two reviewers (YN and KK) independently extracted data from the included studies using a standardized data collection form, Rayyan [13]. Disagreements were discussed, and if they could not be resolved, a third reviewer arbitrated (YK).

Data Items

The collected information encompassed the study context, including the primary author, year of publication, and location; study design; study population (number of participants, age, and procedure type); interventions (remimazolam dose); and outcomes (incidence of ED, emergence time, length of PACU stay, severity of delirium, and all adverse events). Disagreements were discussed, and if they could not be resolved, a third reviewer arbitrated (YK).

Risk of Bias Assessment

Two reviewers (YN and KK) independently reviewed and analyzed the risk of bias using the Risk of Bias 2 tool [14]. Disagreements were discussed, and if they could not be resolved, a third reviewer arbitrated (YK).

Effect Measures

We pooled the relative risk ratios (RRs), risk difference (RD), and 95% confidence intervals (CIs) for the binary variable, incidence of ED. We pooled the mean differences and 95% CIs for the continuous variables: emergence time, severity of delirium, and length of PACU stay. We summarized adverse events based on the definition in the original article, but we did not perform a meta-analysis. When both the intention-to-treat and per-protocol effects were reported, we selected the per-protocol effect.

Dealing With Missing Data

When needed, we requested the study authors to provide data that were not included in their study [11]. A meta-analysis was conducted using the data available from the included studies. For results presented as medians with interquartile ranges, we transformed the data to mean ± standard deviation, as proposed by Wan et al. [15].

Assessment of Heterogeneity

We assessed the statistical heterogeneity by visually examining the forest plots and calculating the I^2^ statistic. The interpretation of I² values was as follows: 0% to 40% was considered “might not be important,” 30% to 60% was interpreted as “may represent moderate heterogeneity,” 50% to 90% was regarded as “may represent substantial heterogeneity,” and 75% to 100% was categorized as “considerable heterogeneity.” In cases where substantial heterogeneity (I² > 50%) was identified, we explored potential sources of variability.

Meta-Analysis

Meta-analysis was conducted using Review Manager software (RevMan 5.4, Cochrane Collaboration, London, United Kingdom), and random-effects models were applied.

Subgroup Analysis

To examine the impact of effect modifiers on the outcomes, we conducted subgroup analyses on the primary outcomes, based on different administration patterns of remimazolam (bolus dosage or continuous dosage) [7-9].

Reporting Bias Assessment

We did an extensive literature search for unpublished trials by searching clinical trial registration systems (ClinicalTrials.gov and ICTRP). We assessed the potential publication bias by searching for the trial registrations through ICTRP and ClinicalTrials.gov and the discrepancies between the reported studies and trial registrations.

Certainty Assessment

Two reviewers (YN and KK) assessed the certainty of the evidence using the Grading of Recommendation, Assessment, Development, and Evaluation (GRADE) approach [16]. Disagreements between the reviewers were resolved through discussion, and if consensus could not be reached, a third reviewer arbitrated (YK). In accordance with the Cochrane Handbook [11], a summary of findings (SoF) table was prepared to summarize the results for the incidence of ED, emergence time, severity of delirium, and length of PACU stay.

Results

Search Results and Characteristics of the Included Trials

**Figure 1:**
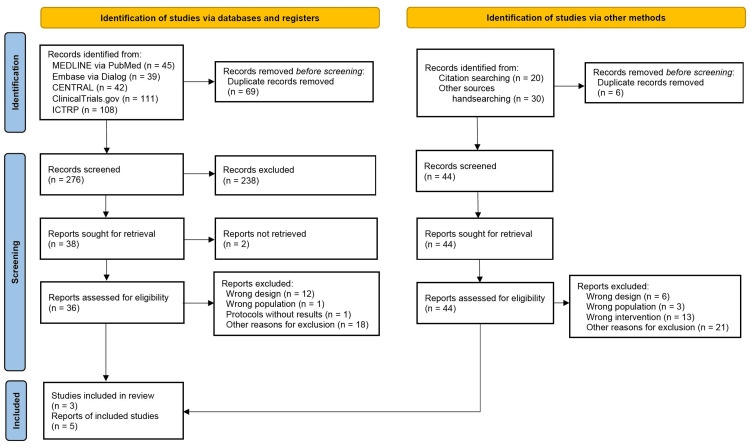
PRISMA 2020 flow diagram for this study CENTRAL: Cochrane Central Register of Controlled Trials; ICTRP: International Clinical Trials Registry Platform; RCTs: randomized controlled trials; PRISMA: Preferred Reporting Items for Systematic Review and Meta-Analysis

After removing duplicates, 276 records were retrieved from database searches conducted on June 30, 2024 (Figure 1). In total, 38 reports were identified, and 36 underwent full-text screening for eligibility. Ultimately, 32 reports were excluded, leaving three studies (n = 310) that met all eligibility criteria, including those identified through citation tracking and manual searches (Appendix C) [7-9].

As summarized in Table 1, all included studies [7-9] were carried out in China, with sample sizes of 90-119 per study. This systematic review included three separate studies, each involving procedures performed under general anesthesia with sevoflurane. These studies included bilateral tonsillectomies and adenoidectomies, laparoscopic inguinal hernia repairs, and dental procedures. All studies involved pediatric patients no older than seven years with an ASA-PS classification of I or II. In all studies, saline was used as a placebo control, and remimazolam was used as an intervention in one or two different ways. One study [7] compared it to a single dose of 0.2 mg/kg administered at the conclusion of the surgery, coinciding with the cessation of sevoflurane and nitrous oxide. Another study [9] compared it to either a single dose of 0.2 mg/kg given approximately five minutes before the end of the surgery or a continuous infusion until approximately five minutes before the end of the surgery. The third study [8] compared it to a single dose of either 0.2 mg/kg or 0.1 mg/kg given about five minutes before the end of the surgery.

**Table 1:** Characteristics of included studies Characteristics of a randomized, placebo-controlled trial evaluating the concomitant use of remimazolam during general anesthesia with sevoflurane to prevent emergence delirium in children. ^†^number of patients enrolled (number of patients included in the analysis) y: year; IV: intravenous infusion; CIV: continuous intravenous infusion; PAED: Pediatric Anesthesia Emergence Delirium score; mYPAS: modified Yale Preoperative Anxiety Scale; PONV: postoperative nausea and vomiting; PACU: post-anesthesia care unit; FLACC: Face, Legs, Activity, Cry, and Consolability; BIS: Bispectral Index

Author	Year	Country	Study design	Number of patients^†^	Age min-max	Description of procedure(s)	Intervention group, n (%)	Control group, n (%)	Maintenance of anesthesia	Primary outcome	Second outcome
Cai et al. [9]	2024	China	Single-center, randomized clinical trial	120 (119)	1-6 y	Laparoscopic inguinal hernia repair	Remimazolam 0.2 mg/kg IV bolus, 39 (32.8) Remimazolam 1 mg/kg/h CIV, 40 (33.6)	Normal saline, 40 (33.6)	Sevoflurane	Emergence delirium	FLACC score, recovery time, the number of rescues of propofol administered in PACU, the end-tidal concentration of sevoflurane to maintain a BIS within 40-60, blood pressure and heart rate, and the incidence of adverse events
Tao et al. [8]	2023	China	Single-center, randomized clinical trial	90 (90)	3-7 y	Tooth decay treatment	Remimazolam 0.1 mg/kg IV bolus, 30 (33.3) Remimazolam 0.2 mg/kg IV bolus, 30 (33.3)	Normal saline, 30 (33.3)	Sevoflurane	Emergence agitation	The mYPAS score, anesthesia time, operation time, extubation time, eye-opening time, PACU stay time, Ramsay sedation score, FLACC score, and adverse reactions (PONV, drowsiness, hypoxia, and increased secretions).
Yang et al. [7]	2022	China	Single-center, randomized clinical trial	104 (101)	3-7 y	Bilateral tonsillectomy and adenoidectomy	Remimazolam 0.2 mg/kg IV bolus, 51 (50.5)	Normal saline, 50 (49.5)	Sevoflurane	Emergence delirium	The peak PAED score, emergence time, postoperative pain intensity, length of PACU stay, parental satisfaction, postoperative behavior changes three days postoperatively, and intraoperative adverse events (PONV, bradycardia, oxygen desaturation, and laryngospasm)

**Figure 2:**
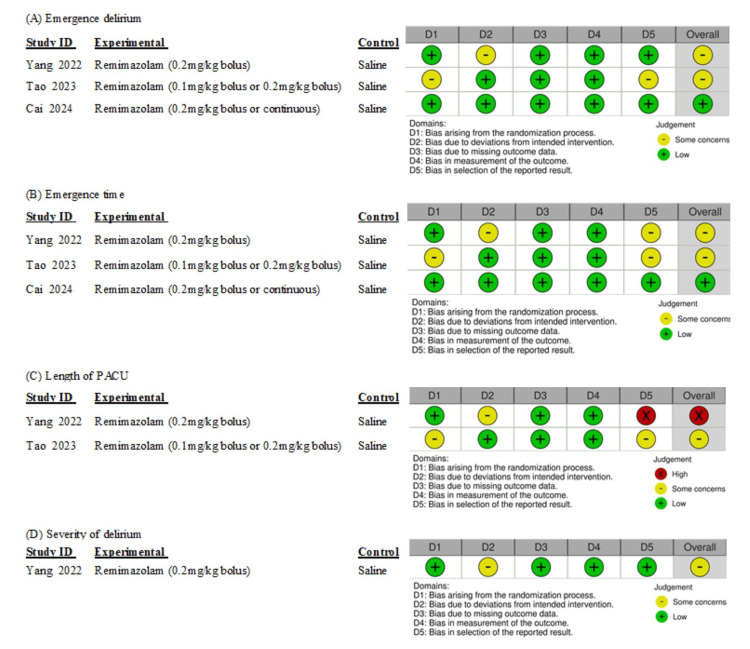
Risk of bias for each outcome in included studies References: [7-9] PACU: post-anesthesia care unit

The risk of bias for each outcome across the studies, which ranged from low to high, is shown in Figure 2. For ED, 33.3% of the assessments indicated a low risk of bias, while 66.6% showed some concerns. Regarding emergence time, 33.3% were categorized as low risk, and 66.6% were identified as having some concerns. For the length of PACU, 50% presented some concerns and 50% were high risk. Regarding delirium severity, the overall risk of bias was low risk. Particularly in the selection of reported results, many studies presented some concerns. We were unable to obtain the protocol data from one author [8].

Primary Outcomes

The SoFs used in this study are listed in Table 2. For more details, see Appendix D.

**Table 2:** Summary of findings Patient or population: pediatric patients (0 to 18 years of age); Setting: pediatric patients who require general anesthesia with inhaled sevoflurane to undergo surgery; Intervention: Remimazolam; Comparison: placebo Explanations a. Downgraded by two levels due to risk of bias (unclear) and small sample size (n = 60). b. Only one study reported a single bolus dose of 0.1 mg/kg remimazolam. As this outcome was measured in only one study, heterogeneity cannot be assessed. c. Downgraded by two levels due to risk of bias (unclear) and small sample size (n = 240). d. Downgraded by two levels due to risk of bias (unclear) and small sample size (n = 80). e. Only one study reported on continuous infusion of remimazolam. As this outcome was measured in only one study, heterogeneity cannot be assessed. f. Downgraded by three levels due to risk of bias (unclear), high heterogeneity (75-100%), and serious imprecision (the confidence interval around the effect included a clinically meaningful effect for either the intervention or the control, and a small sample size (n = 240)). g. Downgraded by two levels due to risk of bias (unclear) and small sample size (n = 80). h. Downgraded by three levels due to risk of bias (high or unclear), high heterogeneity (75-100%), and serious imprecision (the confidence interval around the effect included a clinically meaningful effect for either the intervention or the control, and a small sample size (n = 181)). *The risk in the intervention group (and its 95% confidence interval) is based on the assumed risk in the comparison group and the relative effect of the intervention (and its 95% CI). A hyphen ("-") indicates that no additional comments were provided for the respective entry. CI: confidence interval; MD: mean difference; RR: risk ratio; PACU: post-anesthesia care unit; RCT: randomized controlled trial

Outcomes	Anticipated absolute effects* (95% CI)		Relative effect (95% CI)	No. of participants (studies)	Certainty of the evidence (GRADE)	Comments
	Risk with placebo (normal saline)	Risk with remimazolam				
Emergence delirium - Bolus 0.1 mg/kg	567 per 1,000	232 per 1,000 (113 to 482)	RR 0.41 (0.20 to 0.85)	60 (1 RCT)	⨁⨁◯◯ Low ^a,b^	-
Emergence delirium - Bolus 0.2 mg/kg	475 per 1,000	124 per 1,000 (76 to 209)	RR 0.26 (0.16 to 0.44)	240 (3 RCTs)	⨁⨁◯◯ Low ^c^	-
Emergence delirium - Continuous	450 per 1,000	99 per 1,000 (36 to 270)	RR 0.22 (0.08 to 0.60)	80 (1 RCT)	⨁⨁◯◯ Low ^d,e^	-
Emergence time - Bolus 0.1 mg/kg	The mean emergence time - Bolus 0.1 mg/kg was 0	MD 0.5 higher (0.31 lower to 1.31 higher)	Not estimated	60 (1 RCT)	⨁⨁◯◯ Low ^a,b^	-
Emergence time - Bolus 0.2 mg/kg	The mean emergence time - Bolus 0.2 mg/kg was 0	MD 2.81 higher (1.5 lower to 7.12 higher)	Not estimated	240 (3 RCTs)	⨁◯◯◯ Very low ^f^	-
Emergence time - Continuous	The mean emergence time - Continuous was 0	MD 5.7 higher (3.67 higher to 7.73 higher)	Not estimated	80 (1 RCT)	⨁⨁◯◯ Low ^e,g^	-
Length of PACU - Bolus 0.2 mg/kg	The mean length of PACU - Bolus 0.2 mg/kg was 0	MD 2.01 higher (4.94 lower to 8.97 higher)	Not estimated	161 (2 RCTs)	⨁◯◯◯ Very low ^h^	-
GRADE Working Group grades of evidence: High certainty: we are very confident that the true effect lies close to that of the estimate of the effect. Moderate certainty: we are moderately confident in the effect estimate: the true effect is likely to be close to the estimate of the effect, but there is a possibility that it is substantially different. Low certainty: our confidence in the effect estimate is limited; the true effect may be substantially different from the estimate of the effect. Very low certainty: we have very little confidence in the effect estimate; the true effect is likely to be substantially different from the estimate of the effect.						

**Figure 3:**
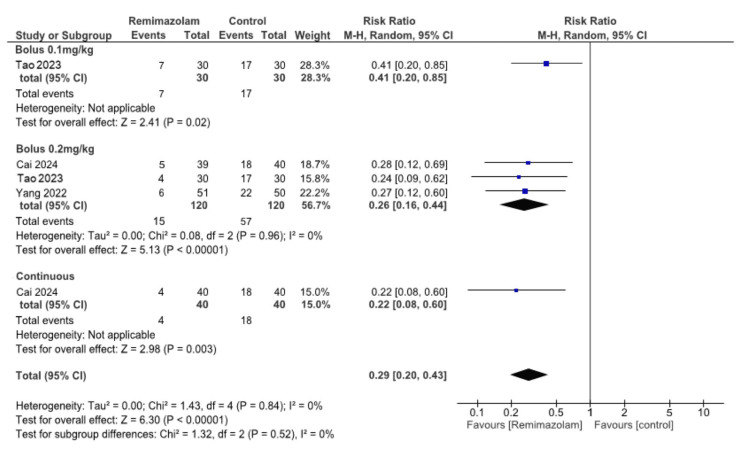
Forest plot for emergence delirium (risk ratio) The square for each study (first author and publication year) represents the risk ratio (RR). The corresponding horizontal line indicates a 95% confidence interval (CI). The upper diamond represents the pooled RR of the subgroup (remimazolam 0.2 mg/kg bolus) with a 95% CI. The lower diamond indicates the pooled RR with the 95% CI. References: [7-9]

**Figure 4:**
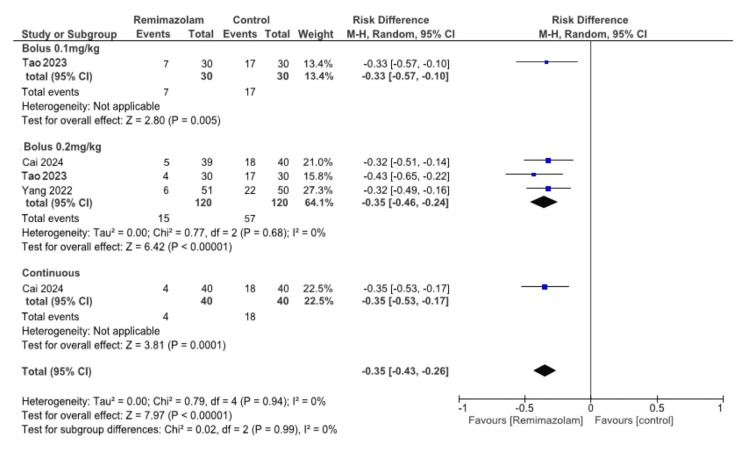
Forest plot for emergence delirium (risk difference) The square for each study (first author and publication year) represents the risk difference (RD) for individual trials. The corresponding horizontal line indicates the 95% CI. The upper diamond represents the pooled RR of the subgroup (remimazolam 0.2 mg/kg bolus) with a 95% CI. The lower diamond indicates the pooled RR with the 95% CI. References: [7-9]

Emergence delirium: A bolus of 0.1 mg/kg remimazolam may reduce ED (one study, 60 participants, RR 0.41, 95% CI: 0.20 to 0.85, RD -0.33, 95% CI: -0.57 to -0.10; low certainty evidence). A bolus of 0.2 mg/kg remimazolam may result in a large reduction in ED (three studies, 240 participants, RR 0.26, 95% CI: 0.16 to 0.44, I² = 0%, RD -0.35, 95% CI: -0.46 to -0.24, I² = 0%; low certainty evidence). Continuous infusion of remimazolam may reduce ED (one study, 80 participants, RR 0.22, 95% CI: 0.08 to 0.60, RD -0.35, 95% CI: -0.43 to -0.26; low certainty evidence) (Figures 3-4).

**Figure 5:**
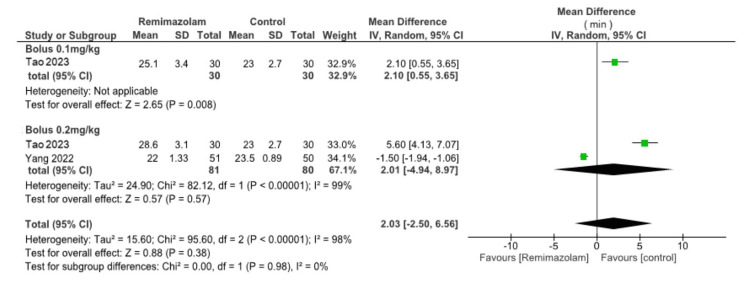
Forest plot for emergence time The square for each study (first author and publication year) represents the mean difference (MD) of individual trials. The corresponding horizontal line indicates the 95% CI. The upper diamond represents the pooled MD of the 0.2 mg/kg remimazolam bolus group with a 95% CI. The lower diamond represents the pooled MD with a 95% CI. SD: standard deviation; CI: confidence interval References: [7-9]

Emergence time: A bolus of 0.1 mg/kg remimazolam may result in little to no difference in emergence time (one study, 60 participants, mean difference (MD) 0.5 min, 95% CI -0.31 to 1.13, heterogeneity could not be assessed; low certainty evidence). The evidence regarding the effect of a 0.2 mg/kg bolus of remimazolam on emergence time is very uncertain (three studies, 240 participants, MD 2.81 min, 95% CI -1.5 to 7.12, I² = 99%; very low certainty evidence). Continuous infusion of remimazolam may result in a slight increase in emergence time (one study, 80 participants, MD 5.7 min, 95% CI 3.67 to 7.73, heterogeneity cannot be assessed; low certainty evidence) (Figure 5).

Secondary Outcomes

Length of PACU: A bolus of 0.1 mg/kg remimazolam may result in a slight increase in PACU stay (one study, 60 participants, MD 2.10 min, 95% CI 0.55 to 3.65, heterogeneity cannot be assessed; low certainty evidence). The evidence is very uncertain about the effect of a 0.2 mg/kg bolus of remimazolam on PACU stay duration (two studies, 161 participants, MD 2.01 min, 95% CI -4.94 to 8.97, I², 99%; very low certainty evidence) (Appendix A).

Severity of delirium: A bolus of 0.2 mg/kg remimazolam may result in a slight reduction in the severity of delirium (one study, 101 participants, MD -2.30, 95% CI -2.52 to -2.08, heterogeneity cannot be assessed; low certainty evidence) (Appendix B).

The PRISMA 2020 checklist for this study is available in Appendix E.

Discussion

In this study, we investigated the concomitant use of intravenous remimazolam with inhalation anesthesia and its effects on ED in children through an SR/MA. We included three RCTs involving 310 pediatric patients. Although evidence regarding the effect of remimazolam on emergence time is very uncertain, both bolus and continuous infusion of remimazolam may result in a reduction in the incidence of ED. The concomitant use of intravenous remimazolam with inhalation anesthesia may be a feasible option, particularly when physicians are concerned about subsequent ED.

Concomitant use of remimazolam and inhaled anesthetics may reduce ED incidence during general anesthesia in children. In pediatric anesthesia, the concomitant use of propofol is considered among the most effective agents for reducing the risk of ED [4,5]. According to a previous SR/MA, a propofol prophylactic dose of 1 mg/kg significantly reduced the incidence of ED when compared to placebo (RR 0.57, 95% CI, 0.43-0.76) [4]. Another SR/MA showed that propofol, administered at doses between 0.5 mg/kg and 3.0 mg/kg, was effective in reducing the incidence of ED (RR 0.51, 95% CI 0.39-0.67) [5]. These results were similar to those obtained in our analysis. Remimazolam may serve as an alternative when propofol is not suitable due to allergies or other concerns. In addition to propofol, other agents have also been reported to decrease the incidence of ED. An SR/MA using ketamine at doses between 0.25 mg/kg and 1 mg/kg demonstrated that ketamine effectively decreased the incidence of ED in pediatric patients (OR 0.23, 95% CI: 0.11-0.46) [17]. Another SR/MA that included various administration routes of dexmedetomidine showed that dexmedetomidine, regardless of the administration route, was highly effective in reducing ED in pediatric patients. (OR 0.22, 95% CI: 0.16-0.32) [18]. In contrast, a previous network meta-analysis indicated that midazolam was less effective than other agents, such as dexmedetomidine and ketamine, and its preventive effect alone was limited [19]. Although direct comparisons between remimazolam and these agents are limited by background differences in dosage, study design, and surgical procedures, remimazolam may offer similar benefits in preventing ED. Further studies are required to directly compare remimazolam to other agents.

In the prevention of ED, careful consideration must be given to both the effectiveness of the intervention and its potential impact on emergence time. Our study found that the effect of remimazolam on emergence time varied with dose and route of administration. While the administration of remimazolam could cause a minor delay in emergence time compared with its absence, this delay might not have a clinical impact. This aligns with findings from prior SR/MA studies of propofol [4,5], which also showed a small delay in emergence time without a clinically meaningful effect. In children, the context-sensitive half-life of remimazolam is approximately 17 minutes following an infusion lasting an hour [20], whereas that of propofol ranges from 10.4 minutes after a one-hour infusion to 19.6 minutes after a four-hour infusion [21], suggesting that the two agents might be considered pharmacologically equivalent. However, the evidence regarding remimazolam’s effect on emergence time was highly uncertain in our review, making it difficult to assess its clinical implications with confidence.

The primary strength of our study is that it is the first SR/MA to evaluate the efficacy of remimazolam in reducing ED after general anesthesia in children with inhaled anesthetics. Furthermore, we conducted an extensive investigation to identify relevant evidence following the PRISMA guidelines [10] and applied the GRADE approach [16] to assess the certainty of the evidence.

This study has several limitations. First, it included only three studies [7-9], leading to a limited dataset. Second, all the studies were conducted within China and focused on pediatric patients without the inclusion of neonates or infants, which may restrict the generalization of results to other countries or different age groups. Healthcare systems and genetic factors may also influence the effects of remimazolam. Hu et al. demonstrated that genetic factors affect the metabolism of midazolam and similar effects could be seen with remimazolam as well [22]. Therefore, further studies in different populations and genetic backgrounds are necessary to confirm these findings. Previous studies have identified multiple risk factors for ED. In pediatric patients, age, anesthesia methods, preoperative anxiety, and pain have been reported as significant contributors [23]. In adults, particularly older adults, preoperative anxiety, inadequate pain management, opioid use, and type of surgery have been associated with an increased risk of ED [24,25]. Additionally, several risk factors for postoperative delirium have been identified, including advanced age, multiple comorbidities, severity of illness, low functional reserve or frailty, and preexisting cognitive impairment [25,26]. While some of these factors were evaluated in the included studies, their assessment was inconsistent and often incomplete, raising concerns about the generalizability of our findings. Future studies should incorporate more comprehensive documentation of these variables to better evaluate their interactions and overall impact on ED outcomes. Finally, the overall risk of bias varied among the included studies, with some showing “some concerns” or “high risk of bias” in the selection of the reported results. Additional high-quality studies with larger sample sizes are necessary to establish definitive conclusions.

## Conclusions

In conclusion, this SR/MA suggests that the concomitant use of remimazolam might decrease the incidence of ED after pediatric anesthesia without a clinically meaningful prolongation of emergence time. Therefore, anesthetists could potentially use remimazolam to reduce ED in children after inhalation anesthesia. While the evidence remains limited, our findings indicate a potential association between remimazolam administration and a reduction in ED incidence. However, variations in study design and sample sizes warrant cautious interpretation. Further, high-quality RCTs are needed to establish definitive conclusions regarding efficacy and safety across diverse populations and to guide their optimal use in clinical practice.

## Figures and Tables

**Figure 6:**
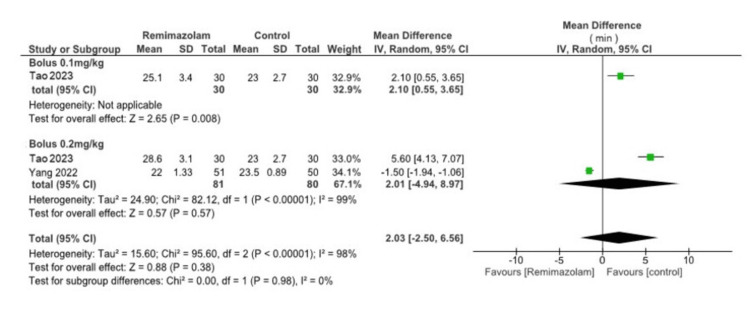
Forest plot for the length of PACU. The square for each study (first author and year of publication) represents the MD of individual trials. The corresponding horizontal line indicates the 95% CI. The upper diamond represents the pooled MD of the 0.2 mg/kg Remimazolam bolus group with a 95% CI. The lower diamond represents the pooled MD with a 95% CI MD: mean difference; PACU: post-anesthesia care unit

**Figure 7:**
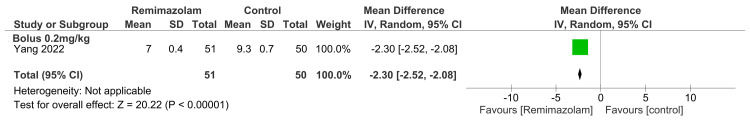
Forest plot for severity of delirium. The square for a study (first author and publication year) represents the MD of individual trials. The corresponding horizontal line indicates the 95% CI. The diamond represents the MD of the 0.2 mg/kg Remimazolam bolus group with a 95% CI MD: mean difference

## Appendices

Appendix A

Appendix B

Appendix C

**Table 3:** Studies excluded from screening

ID	Title	Year	Author	Excluded reasons
1	Postoperative Quality of Recovery After Patients' Undergoing General Anesthesia With Remimazolam Compared to Propofol, A Randomized Non-inferiority Trial	2024	Anshi Wu et al.	Wrong population
2	Investigation into the application of remimazolamin conjunction with low-dose propofolfor pediatricfiberoptic bronchoscopy	2024	Chen W et al.	Wrong study design
3	Clinical study of remimazolam tosilate for bronchoscopy in children	2022	Wang Shaochao et al.	Wrong study design
4	Clinical study on remimazolam besylate for induction and maintenance of anesthesia in children	2023	Gong Yuan et al.	Wrong study design
5	Effect of remimazolam total intravenous general anesthesia on emergence agitation in children undergoing laparoscopic surgery	2023	Huang Ding et al.	Wrong study design
6	Effect of remazolam on root canal recovery in children under combined anesthesia	2023	Na Xing et al.	Protocols without results
7	Pharmacokinetics of remimazolam after intravenous infusion in anaesthetised children	2023	Gao YQ et al.	Wrong study design
8	Remimazolam for Pediatric Procedural Sedation: Results of an Institutional Pilot Program	2023	Hirano T et al.	Wrong study design
9	Anesthetic management with remimazolam for a pediatric patient with Duchenne muscular dystrophy	2021	Horikoshi Y et al.	Other
10	Bibliometric Analysis of Global Trends in Remimazolam-Related Research Over the Past 15 Years: Compared with Propofol	2023	Hu X et al.	Other
11	Remimazolam for Sedation During Fiberoptic Intubation in an Adolescent	2023	Hughes M et al.	Other
12	A case of pediatric Perthes' disease with unexplained hyperlactatemia at the time of initial surgery and anesthetic management with remimazolam for the subsequent surgery	2024	Ishikawa K et al.	Other
13	Successful recording of direct cortical motor-evoked potential from a pediatric patient under remimazolam anesthesia: a case report	2022	Kamata K et al.	Other
14	Pharmacologic Considerations for Pediatric Sedation and Anesthesia Outside the Operating Room: A Review for Anesthesia and Non-Anesthesia Providers	2017	Khurmi N et al	Other
15	Remimazolam as an Adjunct to General Anesthesia in Children: Adverse Events and Outcomes in a Large Cohort of 418 Cases	2023	Kimoto Y et al.	Other
16	A Remimazolam and Remifentanil Anesthetic for a Pediatric Patient With a Medium-Chain Acyl-CoA Dehydrogenase Deficiency: A Case Report	2022	Kiyokawa M et al.	Other
17	Remimazolam for sedation and anesthesia in children: A scoping review	2024	Kuklin V et al	Other
18	Remimazolam and Remifentanil Anesthetics for an Adolescent Patient with Stiff-Person Syndrome: A Case Report	2024	Morita H etaal.	Other
19	Effect of Remimazolam vs Sevoflurane Anesthesia on Incidence of Emergence Agitation and Complications in Children Undergoing Ophthalmic Surgery	2022	Fuzhou Hua, Second Affiliated Hospital of Nanchang University	Wrong study design
20	Comparison of Emergence Delirium: remimazolam vs Sevoflurane Anesthesia	2024	Huacheng Liu et al.	Wrong study design
21	Remimazolam in a Pediatric Patient With a Suspected Family History of Malignant Hyperthermia	2022	Petkus H et al.	Other
22	Effect of Remimazolam and Sevoflurane Anesthesia on Recovery in Pediatric Patients	2024	Hee Young Kim et al.	Wrong study design
23	General Anesthesia With Remimazolam During Minimally Invasive Cardiac Surgery for Atrial Septal Defect: A Pediatric Case Report	2024	Shimizu T et al.	Other
24	Novel anesthetics in pediatric practice: is it time?	2022	Useinovic N et al.	Other
25	General anesthesia with remimazolam for a pediatric patient with MELAS and recurrent epilepsy: a case report	2022	Yamadori Y et al.	Other
26	Clinical Application and Research Progress of Remimazolam for Pediatric Patients	2024	Bai C et al.	Other
27	The safety and efficacy of remimazolam tosylate for induction and maintenance of general anesthesia in pediatric patients undergoing elective surgery: Study protocol for a multicenter, randomized, single-blind, positive-controlled clinical trial	2023	Fang Y et al.	Wrong study design
28	Efficacy of esketamine combined with different doses of remimazolam for induction of general anesthesia in pediatric patients	2024	Ji L et al.	Wrong study design
29	Successful recording of direct cortical motor evoked potential from a pediatric patient under remimazolam anesthesia	2023	Kamata, K et al.	Other
30	Remimazolam for anesthesia and sedation in pediatric patients: a scoping review	2024	Pieri M et al.	Other
31	Remimazolam in Pediatric Anesthesia and Sedation	2023	Ramamurthi R et al.	Other
32	Pharmacodynamics of remimazolam tosilate inducing loss of consciousness when combined with sufentanil in children	2024	Wang S et al.	Wrong study design
33	Remimazolam for sedation and anesthesia in children: Protocol for a scoping review.	2024	Brüggemann V et al.	Other
34	Efficacy and Safety of Remimazolam Tosilate Combined with Propofol in Digestive Endoscopy: A Randomised Trial.	2023	Cui L et al.	Wrong intervention
35	Electroencephalographic insights into the pathophysiological mechanisms of emergence delirium in children and corresponding clinical treatment strategies.	2024	Gao X et al.	Wrong study design
36	Bibliometric Analysis of Global Trends in Remimazolam-Related Research Over the Past 15 Years: Compared with Propofol.	2023	Hu X et al.	Other
37	Remimazolam and serious adverse events: A scoping review.	2023	Kempenaers S et al.	Other
38	Comparison of the Incidence of Postoperative Acute Kidney Injury Following the Administration of Remimazolam or Sevoflurane in Elderly Patients Undergoing Total Knee Arthroplasty: A Randomized Controlled Trial.	2023	Lee S et al.	Wrong population
39	Side effects of sedatives and hypnotics.	2023	Liu MT et al.	Other
40	ED95 of remimazolam in nasal administration for attenuating preoperative anxiety in children.	2023	Long X et al.	Wrong study design
41	The effect of remimazolam-based total intravenous anesthesia versus sevoflurane-based inhalation anesthesia on emergence delirium in children undergoing tonsillectomy and adenoidectomy: study protocol for a prospective randomized controlled trial.	2024	Ma H-Y et al.	Other
42	The influence of delirium on mortality and length of ICU stay and analysis of risk factors for delirium after liver transplantation.	2023	Ma Y et al.	Other
43	Adenotonsillektomi ve Tonsillektomi operasyonlarında preemptif analjezinin derlenme deliryumu üzerine etkisi.	2024	Olgun Keles B et al.	Wrong study design
44	The Role of Remimazolam in Neurosurgery and in Patients With Neurological Diseases: A Narrative Review.	2023	Teixeira MT et al.	Wrong population
45	Clinical anesthetic effect of esketamine on children undergoing tonsillectomy.	2023	Xiang S et al.	Wrong intervention
46	Comparison of dexmedetomidine and a dexmedetomidine-esketamine combination for reducing dental anxiety in preschool children undergoing dental treatment under general anesthesia: A randomized controlled trial.	2023	Xing F et al.	Wrong intervention
47	Remimazolam for the prevention of emergence agitation in adult following nasal surgery under general anesthesia: A randomized controlled study.	2024	Xu Q et al.	Wrong population
48	A Single Dose of Propofol at the End of Surgery for the Prevention of Emergence Agitation in Children Undergoing Strabismus Surgery during Sevoflurane Anesthesia.	2007	Aouad MT et al.	Wrong intervention
49	Comparison of the effects of 0.03 and 0.05 mg/kg midazolam with placebo on prevention of emergence agitation in children having strabismus surgery.	2014	Cho EJ et al.	Wrong intervention
50	Systematic review of the Face, Legs, Activity, Cry and Consolability scale for assessing pain in infants and children: is it reliable, valid, and feasible for use?	2015	Crellin D et al.	Other
51	Preoperative Anxiety, Postoperative Pain, and Behavioral Recovery in Young Children Undergoing Surgery.	2006	Kain ZN et al.	Other
52	The Yale Preoperative Anxiety Scale : How does it compare with a gold standard?	1997	Kain ZN et al.	Other
53	The effect of midazolam administration for the prevention of emergence agitation in pediatric patients with extreme fear and non-cooperation undergoing dental treatment under sevoflurane anesthesia, a double-blind, randomized study.	2019	Kawai M et al.	Wrong intervention
54	Comparison of propofol and fentanyl administered at the end of anaesthesia for prevention of emergence agitation after sevoflurane anaesthesia in children.	2012	Kim M-S et al.	Wrong intervention
55	Preventive effect of ramelteon on emergence agitation after general anaesthesia in paediatric patients undergoing tonsillectomy: a randomised, placebo-controlled clinical trial.	2020	Komazaki M et al.	Wrong intervention
56	Emergence agitation in paediatric day case surgery: A randomised, single-blinded study comparing narcotrend and heart rate variability with standard monitoring.	2021	Larsen LG et al.	Wrong study design
57	A Review in Procedural Sedation.	2021	Lee A et al.	Other
58	Time-to-Event Modeling for Remimazolam for the Indication of Induction and Maintenance of General Anesthesia.	2020	Lohmer LRL et al.	Other
59	Characterizing the behavior of children emerging with delirium from general anesthesia.	2011	Malarbi S et al.	Other
60	Paediatric emergence delirium: a comprehensive review and interpretation of the literature.	2017	Mason KP:	Other
61	Good Clinical Practice Guidance and Pragmatic Clinical Trials: Balancing the Best of Both Worlds.	2016	Mentz RJ et al.	Other
62	Emergence Delirium in Pediatric Anesthesia.	2016	Moore AD et al.	Wrong intervention
63	Efficacy of 0.5 mg/kg of propofol at the end of anesthesia to reduce the incidence of emergence agitation in children undergoing general anesthesia with sevoflurane.	2020	Ramlan AAW et al.	Wrong intervention
64	CONSORT 2010 Statement: updated guidelines for reporting parallel group randomised trials.	2010	Schulz KF et al.	Other
65	The effect of sub-Tenon lidocaine injection on emergence agitation after general anaesthesia in paediatric strabismus surgery.	2011	Seo I-S et al.	Wrong study design
66	Dexmedetomidine for the prevention of emergence delirium and postoperative behavioral changes in pediatric patients with sevoflurane anesthesia: a double-blind, randomized trial.	2019	Shi M et al.	Wrong intervention
67	Development and psychometric evaluation of the pediatric anesthesia emergence delirium scale.	2004	Sikich N et al.	Other
68	Emergence delirium or pain after anaesthesia—how to distinguish between the two in young children: a retrospective analysis of observational studies.	2016	Somaini M et al.	Other
69	Pharmacokinetic properties of remimazolam in subjects with hepatic or renal impairment.	2021	Stöhr T et al.	Wrong study design
70	Emergence Delirium in Perioperative Pediatric Care: A Review of Current Evidence and New Directions.	2020	Urits I et al:	Wrong intervention
71	Changes in children's behavior after hospitalization. Some dimensions of response and their correlates.	1966	Vernon DTA et al.	Other
72	Excitation and delirium during sevoflurane anesthesia in pediatric patients.	2002	Veyckemans F	Other
73	A prospective cohort study of emergence agitation in the pediatric postanesthesia care unit.	2003	Voepel-Lewis T et al.	Other
74	Discharge readiness after remimazolam versus propofol for colonoscopy: A randomised, double-blind trial.	2022	Yao Y et al	Wrong population
75	Intranasal dexmedetomidine versus oral midazolam premedication to prevent emergence delirium in children undergoing strabismus surgery: A randomised controlled trial.	2020	Yao Y et al	Wrong intervention

Appendix D

**Table 4:** GRADE evidence profile a. Downgraded by one level for risk of bias (unclear risk of bias). b. Only one study reported a bolus dose of 0.1 mg/kg of remimazolam. As this outcome was measured in only one study, the heterogeneity could not be assessed. c. Downgraded by one level for serious imprecision: small sample size (n = 60) d. Downgraded by one level for risk of bias (unclear risk of bias) and one level for imprecision (small sample size (n = 60)) e. Downgraded by one level for risk of bias (two of the three studies had an unclear risk of bias). f. Downgraded by one level for serious imprecision (small sample size (n = 240)) g. Downgraded by one level for risk of bias (unclear risk of bias) and one level for imprecision (small sample size (n = 240)). h. Only one study reported continuous infusion of remimazolam. As this outcome was measured in only one study, the heterogeneity could not be assessed. i. Downgraded by one level for serious imprecision: small sample size (n = 80) j. Downgraded by one level for risk of bias (unclear risk of bias) and one level for imprecision (small sample size (n = 80)). k. Downgraded by one level for serious inconsistency: statistical heterogeneity was 75‐100%. l. Downgraded by two levels for serious imprecision: the confidence interval around the effect included a clinically meaningful effect with the intervention or control and a small sample size (n = 240). m. Downgraded by one level for risk of bias (unclear risk of bias) and two levels for imprecision. n. Downgraded by one level for risk of bias (unclear risk of bias) and one level for imprecision (small sample size (n = 80)). o. Downgraded by two levels of risk of bias (one study was at a high risk of bias and the other had an unclear risk of bias). p. Downgraded by two levels for serious imprecision: the confidence interval around the effect included a clinically meaningful effect with intervention or control and a small sample size (n = 181). MD: mean difference; PACU: post-anesthesia care unit

Certainty assessment								Summary of findings			
Risk of bias	Inconsistency	Indirectness	Imprecision	Publication bias	Overall certainty of evidence	Study event rates (%)			Relative effect	Anticipated absolute effects	
						With placebo（normal saline）	With Remimazolam		(95% CI)	Risk with placebo	Risk difference with Remimazolam
Emergence delirium - Bolus 0.1mg/kg											
serious^a^	not applicable^b^	not serious	serious^c^	none	⨁⨁◯◯	17/30 (56.7%)	7/30 (23.3%)		RR 0.41	17/30 (56.7%)	334 fewer per 1,000
					Low^ d^				(0.20 to 0.85)		(from 453 fewer to 85 fewer)
Emergence delirium - Bolus 0.2mg/kg											
serious^e^	not serious	not serious	serious^f^	none	⨁⨁◯◯	57/120 (47.5%)	15/120 (12.5%)		RR 0.26	57/120 (47.5%)	352 fewer per 1,000
					Low^g^				(0.16 to 0.44)		(from 399 fewer to 266 fewer)
Emergence delirium - Continuous											
serious^a^	not applicable^h^	not serious	serious^i^	none	⨁⨁◯◯	18/40 (45.0%)	4/40 (10.0%)		RR 0.22	18/40 (45.0%)	351 fewer per 1,000
					Low^ j^				(0.08 to 0.60)		(from 414 fewer to 180 fewer)
Emergence time - Bolus 0.1mg/kg											
serious^a^	not applicable^b^	not serious	serious^c^	none	⨁⨁◯◯	30	30		-	30	MD 0.5 higher
					Low^ d^						(0.31 lower to 1.31 higher)
Emergence time - Bolus 0.2mg/kg											
serious^e^	serious^k^	not serious	very serious^l^	none	⨁◯◯◯	120	120		-	120	MD 2.81 higher
					Very low^m^						(1.5 lower to 7.12 higher)
Emergence time - Continuous											
serious^a^	not applicable^h^	not serious	serious^i^	none	⨁⨁◯◯	40	40		-	40	MD 5.7 higher
					Low^ n^						(3.67 higher to 7.73 higher)
Length of PACU - Bolus 0.2mg/kg											
very serious^o^	serious^k^	not serious	very serious^p^	none	⨁◯◯◯	80	81		-	80	MD 2.01 higher
					Very low						(4.94 lower to 8.97 higher)

Appendix E

**Table 5:** PRISMA 2020 check list PRISMA: Preferred Reporting Items for Systematic Review and Meta-Analysis

Section and topic	Item no.	Checklist item	Location where item is reported
TITLE			
Title	1	Identify the report as a systematic review.	OK
ABSTRACT			
Abstract	2	See the PRISMA 2020 for Abstracts checklist.	OK
INTRODUCTION			
Rationale	3	Describe the rationale for the review in the context of existing knowledge.	Page 5, Line 5-
Objectives	4	Provide an explicit statement of the objective(s) or question(s) the review addresses.	Page 5, Line 24-
METHODS			
Eligibility criteria	5	Specify the inclusion and exclusion criteria for the review and how studies were grouped for the syntheses.	Page 6, Line 8-
Information sources	6	Specify all databases, registers, websites, organisations, reference lists and other sources searched or consulted to identify studies. Specify the date when each source was last searched or consulted.	Page 7, Line 16-
			Page 10, Line 17
Search strategy	7	Present the full search strategies for all databases, registers and websites, including any filters and limits used.	Page 7, Line 21
			Appendix 1
Selection process	8	Specify the methods used to decide whether a study met the inclusion criteria of the review, including how many reviewers screened each record and each report retrieved, whether they worked independently, and if applicable, details of automation tools used in the process.	Page 7, Line 27-
Data collection process	9	Specify the methods used to collect data from reports, including how many reviewers collected data from each report, whether they worked independently, any processes for obtaining or confirming data from study investigators, and if applicable, details of automation tools used in the process.	Page 8, Line 7
Data items	10a	List and define all outcomes for which data were sought. Specify whether all results that were compatible with each outcome domain in each study were sought (e.g. for all measures, time points, analyses), and if not, the methods used to decide which results to collect.	Page 6, Line 26-
	10b	List and define all other variables for which data were sought (e.g. participant and intervention characteristics, funding sources). Describe any assumptions made about any missing or unclear information.	Page 8, Line 12-
Study risk of bias assessment	11	Specify the methods used to assess risk of bias in the included studies, including details of the tool(s) used, how many reviewers assessed each study and whether they worked independently, and if applicable, details of automation tools used in the process.	Page 8, Line 19-
Effect measures	12	Specify for each outcome the effect measure(s) (e.g. risk ratio, mean difference) used in the synthesis or presentation of results.	Page 8, Line 24-
Synthesis methods	13a	Describe the processes used to decide which studies were eligible for each synthesis (e.g. tabulating the study intervention characteristics and comparing against the planned groups for each synthesis (item #5)).	Page 7, Line 27-
	13b	Describe any methods required to prepare the data for presentation or synthesis, such as handling of missing summary statistics, or data conversions.	Page 9, Line 5-
	13c	Describe any methods used to tabulate or visually display results of individual studies and syntheses.	Page 9, Line 19-
	13d	Describe any methods used to synthesize results and provide a rationale for the choice(s). If meta-analysis was performed, describe the model(s), method(s) to identify the presence and extent of statistical heterogeneity, and software package(s) used.	Page 9, Line 11-
			Page 9, Line 19-
	13e	Describe any methods used to explore possible causes of heterogeneity among study results (e.g. subgroup analysis, meta-regression).	Page 9, Line 23-
	13f	Describe any sensitivity analyses conducted to assess robustness of the synthesized results.	Not applicable
Reporting bias assessment	14	Describe any methods used to assess risk of bias due to missing results in a synthesis (arising from reporting biases).	Page 10, Line 1-
Certainty assessment	15	Describe any methods used to assess certainty (or confidence) in the body of evidence for an outcome.	Page 10, Line 7-
RESULTS			
Study selection	16a	Describe the results of the search and selection process, from the number of records identified in the search to the number of studies included in the review, ideally using a flow diagram.	Page 10, Line 16-
			Figure 1
	16b	Cite studies that might appear to meet the inclusion criteria, but which were excluded, and explain why they were excluded.	Page 10, Line 18-
			Supplementary Table 1.
Study characteristics	17	Cite each included study and present its characteristics.	Page 10, Line 21-
			Page 19, Table 1.
Risk of bias in studies	18	Present assessments of risk of bias for each included study.	Page 11, Line 7-
			Page 20, Table 2.
Results of individual studies	19	For all outcomes, present, for each study: (a) summary statistics for each group (where appropriate) and (b) an effect estimate and its precision (e.g. confidence/credible interval), ideally using structured tables or plots.	Table 3,
			Figure 2A and 2B,
			Figure 3, Supplementary Figure 1,
			Supplementary Figure 2
Results of syntheses	20a	For each synthesis, briefly summarise the characteristics and risk of bias among contributing studies.	Page 11, Line 7-
			Table 2
	20b	Present results of all statistical syntheses conducted. If meta-analysis was done, present for each the summary estimate and its precision (e.g. confidence/credible interval) and measures of statistical heterogeneity. If comparing groups, describe the direction of the effect.	Page 11, Line 19-
			Table3,
			Figure 2A and 2B,
			Figure 3, Supplementary Figure 1,
			Supplementary Figure 2
	20c	Present results of all investigations of possible causes of heterogeneity among study results.	Page 11, Line 20-
	20d	Present results of all sensitivity analyses conducted to assess the robustness of the synthesized results.	Not applicable
Reporting biases	21	Present assessments of risk of bias due to missing results (arising from reporting biases) for each synthesis assessed.	Page 10, Line 1-
Certainty of evidence	22	Present assessments of certainty (or confidence) in the body of evidence for each outcome assessed.	Table 3
DISCUSSION			
Discussion	23a	Provide a general interpretation of the results in the context of other evidence.	Page 13, Line 7-
	23b	Discuss any limitations of the evidence included in the review.	Page 14, Line 18-
	23c	Discuss any limitations of the review processes used.	Not applicable
	23d	Discuss implications of the results for practice, policy, and future research.	Page 13, Line 5-
OTHER INFORMATION			
Registration and protocol	24a	Provide registration information for the review, including register name and registration number, or state that the review was not registered.	Page 6, Line 3-
	24b	Indicate where the review protocol can be accessed, or state that a protocol was not prepared.	Page 6, Line 3-
	24c	Describe and explain any amendments to information provided at registration or in the protocol.	Not applicable
Support	25	Describe sources of financial or non-financial support for the review, and the role of the funders or sponsors in the review.	Page 15, Line 16
Competing interests	26	Declare any competing interests of review authors.	Page 15, Line 12
Availability of data, code and other materials	27	Report which of the following are publicly available and where they can be found: template data collection forms; data extracted from included studies; data used for all analyses; analytic code; any other materials used in the review.	Page 15, Lie 10-

Appendix F

S1 File 1: Search strategy

CENTRAL search strategy

(((([mh Anesthesia]) OR ([mh Anesthetics])) OR (Anesthe*:ti,ab)) AND (((((((([mh Adolescent]) OR ([mh Infant])) OR ([mh child*])) OR ([mh pediatric])) OR (Adolesce*:ti,ab)) OR (Infan*:ti,ab)) OR (child*:ti,ab)) OR (pediat*:ti,ab))) AND (((((((remimazolam:ti,ab) OR ("CNS 7056":ti,ab)) OR ("ONO 2745":ti,ab)) OR (ONO-2745:ti,ab)) OR (ANEREM:ti,ab)))

MEDLINE (via PubMed) search strategy

#1 Anesthesia[mh]

#2 Anesthetics[mh]

#3 Anesthe*[tiab]

#4 Adolescent[mh]

#5 Infant[mh]

#6 child[mh]

#7 pediatric[mh]

#8 Adolesce*[tiab]

#9 Infan*[tiab]

#10 child*[tiab]

#11 pediat*[tiab]

#12 (#1 OR #2 OR #3) AND (#4 OR #5 OR #6 OR #7 OR #8 OR #9 OR #10 OR #11)

#13 remimazolam[tiab]

#14 CNS 7056[tiab]

#15 ONO 2745[tiab]

#16 ONO-2745[tiab]

#17 ANEREM[tiab]

#18 #13 OR #14 OR #15 OR #16 OR #17

#19 #12 AND #18

EMBASE (via OVID) search strategy

#1 'anesthesia'/exp

#2 'anesthetic agent'/exp

#3 anesthe*:ab,ti

#4 'adolescent'/exp

#5 'infant'/exp

#6 'child'/exp

#7 'pediatric'/exp

#8 adolesce*:ab,ti

#9 infant*:ab,ti

#10 child*:ab,ti

#11 pediat*:ab,ti

#12 #1 OR #2 OR #3

#13 #4 OR #5 OR #6 OR #7 OR #8 OR #9 OR #10 OR #11

#14 #12 AND #13

#15 remimazolam:ab,ti

#16 'cns 7056 ':ab,ti

#17 'ono 2745':ab,ti

#18 ' ono-2745':ab,ti

#19 anerem:ab,ti

#20 #15 OR #16 OR #17 #18 OR #19

#21 #14 AND #20

ICTRP search strategy

(“remimazolam” OR “CNS 7056” OR “ONO 2745” OR “ONO-2745” OR “ONO2747” OR “ANEREM” OR "Remimazolam Besilate") AND ((“Anesthesia” OR “Anesthetics”) AND (“Child” OR “Adolescent” OR “Infant” OR “child*” OR “pediatric”))

ClinicalTrials.gov search strategy

Condition or disease: “Anesthesia” OR “Anesthetics” AND “Child” OR “Adolescent” OR “Infant” OR “child*” OR “pediatric”

Intervention: “remimazolam” OR “CNS 7056” OR “ONO 2745” OR “ONO-2745” OR “ONO2747” OR “ANEREM” OR "Remimazolam Besilate"

Appendix G

The systematic review protocol template is available at the following link: dx.doi.org/10.17504/protocols.io.biqrkdv6.
